# Promoting diversity in geoheritage evaluation: creating an evaluation method for the scientific value of Quaternary sites in arid environments

**DOI:** 10.1098/rsta.2023.0138

**Published:** 2024-04-01

**Authors:** Kenta Sayama

**Affiliations:** School of Geography and the Environment, University of Oxford, Oxford, UK

**Keywords:** geoheritage, Quaternary science, geoconservation, evaluation, arid environments, Middle East-North Africa

## Abstract

A rigorous assessment is an essential part of geoconservation, and choosing an appropriate evaluation method is essential for this process. Globally, an increasing number of sites are being assessed for their geoheritage values, but the most popular methods have been created by researchers with experiences centred in Europe, without considerations of regional differences. To understand whether regional perspectives are required in the evaluation process, this study developed a new method targeting the scientific value of Quaternary geoheritage sites in arid environments, using input from 49 researchers in geosciences and related disciplines with interests in arid landscapes. The results demonstrated the need for a new or modified method, given the different preferences in the weighting system and the necessity to include additional criteria specific to the type of sites targeted by the study. The strong preference to include a new criterion on connections with archaeology/anthropology highlighted the significance of the interdisciplinary scientific values of Quaternary geoheritage sites in arid environments. These findings imply the need for regional diversity or adjustments in geoheritage evaluation. Future research is required to consider such differences for geoheritage values beyond the scientific dimension, such as educational values and touristic values in diverse geographical settings.

This article is part of the Theo Murphy meeting issue 'Geodiversity for science and society'.

## 1. Introduction

To protect and celebrate geoheritage sites in a world with limited resources, their evaluation is essential to select those with the highest need for conservation. Evaluation is identified as one of the primary tasks for geoheritage researchers [1], as it requires a delicate consideration of combining intrinsic values as well as human focused values (economic/touristic, educational, scientific, etc.). Objectivity and transparency are desired traits in a robust evaluation process [1,2], although a level of subjectivity is inevitable for any evaluation. The need for an effective evaluation is evident from the number of methods proposed in the last few decades [3–10], corresponding to the growth of global interest in geoconservation. Of these methods, several popular ‘schools’ have been identified [11], and the most popular methods have been applied to geoheritage sites around the world. In the process of this development, specialized methods for different types of geoheritage sites have been considered, but very few methods have been created for assessing geoheritage sites from specific environmental regions. Focusing on Quaternary geoheritage sites in arid environments, this study presents an exploratory analysis of the necessity of environment-specific methods for geoheritage evaluation and presents a potential model for creating them.

### (a) History of geoheritage evaluation

In this paper, the definition of the term geoheritage by Brocx & Semeniuk [12, p. 62], ‘[g]lobally, nationally, state-wide, to local features of geology, such as its igneous, metamorphic, sedimentary, stratigraphic, structural, geochemical, mineralogic, palaeontologic, geomorphic, pedologic, and hydrologic attributes, at all scales, that are intrinsically important sites, or culturally important sites, that offer information or insights into the formation or evolution of the Earth, or into the history of science, or that can be used for research, teaching, or reference’ will be used. Some researchers such as Brilha [9] advocate for a restricted use of the term geoheritage to sites that present significant science value, and have promoted the use of the term geosites for sites without scientific significance. A more focused definition has its advantages, such as promoting geological sites for values beyond aesthetics. This approach, however, limits the scope of geological heritage in ways contrary to international frameworks of natural heritage, such as the World Heritage Convention [13]. In the World Heritage Convention, natural heritage sites are valued not only from a scientific perspective, but also from aesthetic (natural beauty) and conservation perspectives. Moreover, with the recognition of cultural landscapes, the cultural values of natural heritage sites are acknowledged. With a wider scope of heritage values in mind, an inclusive definition of geoheritage will be used in this paper.

The first systematic evaluation of geoheritage sites was conducted in 1977 by the British Nature Conservancy Council [14]. Although this was a noble effort to provide clarity to the process of selecting geological sites worthy of protection, the lack of a rubric that describes how a site is evaluated meant that the evaluation was dependent on the evaluator's opinion, resulting in a high level of subjectivity. Similarly, the earliest evaluation methods relied solely on the knowledge of the evaluator, leaving the process more or less a ‘black box’ [15].

Since the late 1990s, many geoheritage evaluation methods have been developed. One category of these methods is qualitative methods like that of Brocx & Semeniuk [16], which have advantages of being more descriptive and in assessing sites that require highly nuanced assessments, such as when the site possesses little scientific value, but very high cultural value [12]. On the other hand, many other methods have employed a quantitative and/or semi-quantitative approach with an accompanying rubric that identifies how to evaluate each criterion (or indicator) clearly with a numerical outcome that enables comparisons between assessed sites [2,17]. These methods are not devoid of subjectivity, as many criteria (e.g. aesthetics, rarity, historical significance, cultural significance) used in the evaluation cannot be applied purely on a numerical basis [9]. Nevertheless, quantitative methods have been used more often in geoheritage studies, as they offer relatively higher levels of transparency, comparability and reproducibility [18]. These benefits are especially important as they make the outcomes more straightforward and credible for the interpretation and understanding by non-specialists such as local stakeholders and government officials. Following this recent trend in the development of the scholarship, this paper will focus on quantitative and/or semi-quantitative methods for evaluating geoheritage sites.

Along with the development of overall evaluations of geoheritage sites, many methods have been developed for the evaluation of geotourism potential/value (e.g. [19,20]) and geodiversity (e.g. [7,21]). A more detailed discussion on this topic can be found in papers such as Bruschi & Cendrero [17] (geoheritage evaluation), KubalÌková [19] (geotourism) and Zwoliński *et al*. [21] (geodiversity assessment). In many of these methods, various dimensions (large categories) of geoheritage values are considered. Dimensions of geoheritage values include: scientific value, educational value, touristic value, endangerment status, aesthetic value and cultural value. A number of researchers [1,9,19] consider the scientific value as the central aspect of geoheritage evaluation, as it provides a relatively objective and transparent description of the intrinsic quality of the site. The scientific value is important, as it is the geoscientific elements of sites that distinguishes geoheritage sites from other types of heritage sites. In fact, as mentioned by Erhartič [22], many of the early assessment methods focused primarily on the scientific value. Hence, although it is true that the consideration of other aspects of geoheritage sites is essential, this study will focus on the scientific value.

To assess the scientific value quantitatively, various relevant criteria (factors used to evaluate each dimension) are considered. Table 1 summarizes the criteria used in previous literature to evaluate the scientific value of geoheritage sites. As shown in table 1, many criteria are shared among different methods. Although the criteria used in these methods tend to overlap, their weighting strategy varies. In a quantitative assessment, the weighting is an important consideration, as it determines two things: the relative importance of different criteria towards a specific value dimension of a site and the relative importance of each value dimension towards the overall geoheritage value of a site. However, as mentioned by Zorlu & Dede [25], strategies for weighting have not often been studied in the geoheritage literature. Although it is an essential component of a quantitative assessment to provide a robust outcome, weighting in most methods [5,9,23,24] has simply been created by the professional experience of the researcher(s), which by nature tend to be subjective. This *status quo* contradicts with the need for subjectivity in the assessment process. Other than the researcher's experience, methods for weighting include, equal weighting [5,26] or using the mean value obtained from a survey conducted towards a larger group of relevant regional experts [15]. Of these three methods, the last option, as used by Bruschi *et al.* [15] offers the least subjectivity, by reflecting the opinions of various professionals. When speedy decisions are required, or only a few relevant experts are available for consultation, the use of other methods could be justifiable, but a weighting system derived from the experience of a single or a few researchers inevitably includes inherent subjectivity, which should be minimized in the process of evaluation.

**Table 1.** Summary of criteria used in previous methods for the evaluation of scientific value for geoheritage sites, with criteria developed for this study. The list includes all criteria included in the questionnaire.

criteria	methods in which the criterion was used
representativeness: whether the site demonstrates exemplary characteristics of the geological/geomorphological elements or processes	Panizza [4], Coratza & Giusti [3], Reynard *et al.* [5], Pereira & Pereira [23], De Lima *et al.* [8], Bruschi *et al.* [15], Brilha [9]
integrity: current state of conservation of the main geological/geomorphological elements of the site	Reynard *et al.* [5], Pereira & Pereira [23], KubalÌková [19], Brilha [9], Ruban *et al.* [24]
geological diversity: whether the site and its surrounding area covers a diverse array of geological/geomorphological features of scientific interest	De Lima *et al.* [8], Pereira & Pereira [23], KubalÌková [19], Brilha [9], Ruban *et al.* [24]
rarity: whether the feature is rare or unique in the region, the country or the world	Coratza & Giusti [3], Reynard *et al.* [5], Pereira & Pereira [23], Bruschi *et al.* [15], KubalÌková [19], Brilha [9], Ruban *et al.* [24]
historical value: importance of the site for the development of the field	Coratza & Giusti [3], Reynard *et al.* [5]
scientific knowledge: the extent of scientific knowledge gained from the site	Coratza & Giusti [3], Pereira & Pereira [23], Bruschi *et al.* [15], KubalÌková [19], Brilha [9], Ruban *et al.* [24]
potential for future research: whether there is ongoing research at the site that may lead to new scientific knowledge or if there is specific mention about the need for further research at the site	Coratza & Giusti [3]
connection with other disciplines: whether the site has connections with disciplines other than archaeology/evolutionary anthropology (flood management, natural disaster management, current land use, etc.)	Bruschi *et al.* [15]
chronological resolution: how far back the chronology from the site extends and at what resolution (intervals)	developed by author for this study
connection with the regional archaeology/anthropology: whether the site has significant meaning towards the regional archaeology/anthropology	developed by author for this study

For the weighting method to combine assessments of different value dimensions into one overall metric, strategies from previous studies include: no categorization of different value dimensions [15], the sum of all value dimension [6,10,27] or the use of a matrix [23]. On the other hand, some methods [5,9,28] refrain from calculating an overall value and opt to produce separate outputs for each value dimension as the final outcome. This approach takes into consideration the idea that the objective of the site's conservation and the plan for its use would change the way different value dimensions are prioritized. For example, if a site or an area is to become protected for solely touristic use, incorporating educational values in its overall assessment is unnecessary. While the single output approaches provide simplicity in ranking sites, the multi-output methods offer better flexibility in their application, accounting for different objectives for protecting geoheritage sites.

### (b) Geographical perspectives on geoheritage evaluation

**Figure 1.**
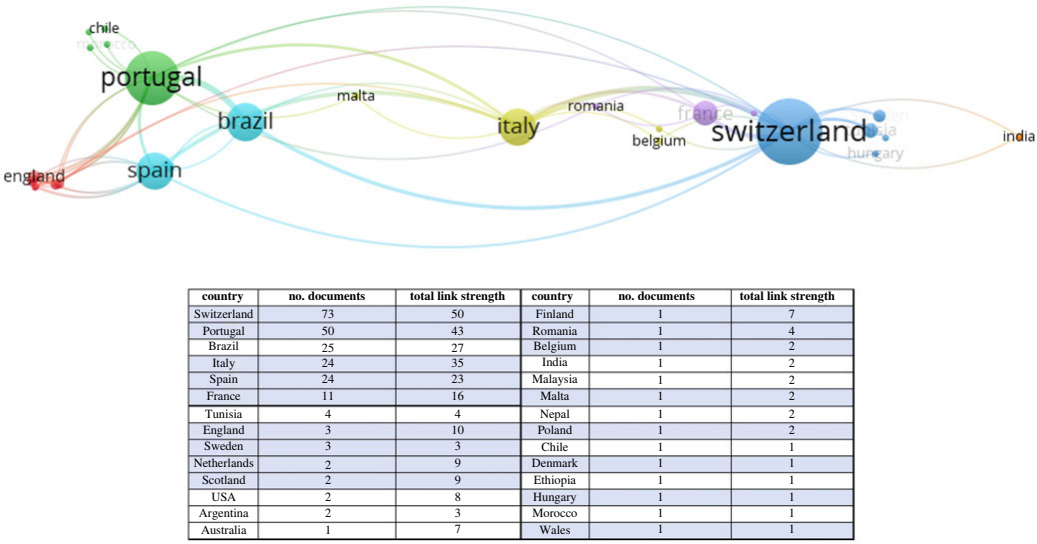
The network of countries where researchers who have co-authored with the first authors of Brilha [9], Bruschi *et al.* [15], Pereira and Pereira [26] and Reynard *et al.* [5] are based (visualization created using VOSviewer with citation data from Web of Science), with a table providing the breakdown of countries included in the network (European countries highlighted in blue). The network includes home countries of the researchers (Portugal, Spain and Switzerland). (Online version in colour.)

In adopting methodologies for geoheritage evaluation, an important consideration is the applicability of the method to different geoheritage sites. Geographical region is an important factor to consider in this regard. Evaluation methods have been developed around the world, such as in Australia by Brocx & Semeniuk [16,29], in Turkey by Zorlu & Dede [25], in Japan by Suzuki & Takagi [30], in Korea by Woo & Kim [31] and in Brazil by Santos *et al*. [32]. The geographical diversity of assessment methods has increased considerably in the last 5 years. Most commonly used methods, however, have been developed in Europe, such as the three methods from Switzerland [5], Spain [15,33] and Portugal [26] identified as the popular schools of geoheritage evaluation by Erhartič [22]. These methods were developed for use at various settings based on the experiences of researchers from some of the pioneering countries for geoheritage research. Furthermore, the method by Brilha [9] has become one of the most cited and applied methods recently, most likely due to its development through a rigorous review of previous methods. In terms of geographical representativeness, these four popular methods for geoheritage evaluation have been developed with experiences predominantly in Europe. As shown in figure 1, creators of these methods not only work in European institutions, but also, most of their collaborators are concentrated in Europe, apart from a cluster in Brazil. This is not to say that they have not conducted work in other regions or lack knowledge on other regions, but it provides an indicator of the Euro-centric foundation of the methods used commonly around the world.

These methods may provide a useful base for conducting geoheritage evaluation around the world. However, they may not be as effective in non-European settings, not only because different regions present different types of sites, but also because the assessment of heritage values requires a human perspective, as can be seen from the inclusion of the cultural value and potential threats/degradation risk in the assessment. Even for the scientific value, the evaluation cannot be based solely on scientific observations, especially when traditional and/or indigenous perspectives toward science or knowledges are considered. As Sillitoe [34] and Whyte *et al.* [35] explain in the context of international development and sustainability science, respectively, respecting, learning and incorporating ideas from different knowledge systems produce better results when working with indigenous peoples.

Zorlu & Dede's [25] assessment of the geoheritage values of palaeoglacial sites in Northern Turkey exemplifies the need for such a regional adjustment. By using opinions by three experts and a suite of statistical techniques, this study considered the need to modify the weighting system of the evaluation method proposed by Ruban & Sallam [24] for application in the study's targeted area. The resulting system diverges significantly from that of the original method in ways such as a lower weight for rarity (66.7–30.8%), and higher weights for scientific importance (3.3–17.8%) and educational importance (3.3–14.6%). The modified weight system helped differentiate the ranking of three sites with identical score in the original method, showing the benefits of a specialized method and potential limitations of evaluation methods designed for global application. Although a universal method for evaluation may be ideal for consistency, adjustments toward these methods should be considered to account for region-specific conditions, such as different climate, geological/geomorphological processes and cultural conditions, when using them in different regions.

### (c) Geoheritage evaluation in arid environments

**Figure 2.**
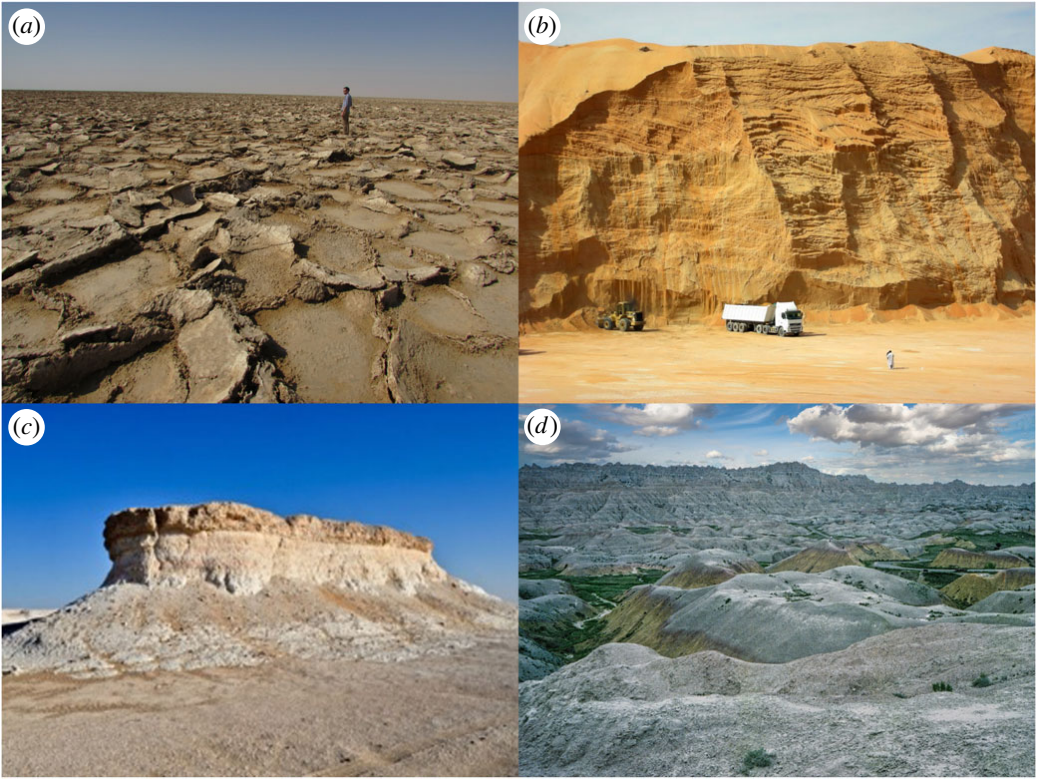
Examples of Quaternary geoheritage from arid environments: (*a*) inland sabkha from Oman, (*b*) section of a mega sand dune in the UAE, (*c*) wind-eroded sandstone from a palaeolake in the UAE, (*d*) badland landscape in southwest USA (Photos courtesy of Prof. Andrew Goudie, Dr Ash Parton and Tom Fink). (Online version in colour.)

Arid environments encompass a significant portion of the Earth's land surface, constituting approximately 41% of its total area [36]. Within arid environments, there are diverse geomorphological characteristics and climatic heterogeneity, but the relative importance of processes and their magnitude and frequency are distinct from other regions, which justifies such a regional categorization from a geoscientific perspective [37]. These distinct processes create characteristic landforms such sand dunes, mesas, sabkhas and palaeolakes/rivers (figure 2 for examples), many of which demonstrate high geoheritage values.

Over the past decade, there has been a notable increase in the number of studies focused on geoheritage evaluation in arid environments, particularly in the MENA (Middle East-North Africa) region. This surge is especially evident in countries such as Iran [11,38–50], Egypt [24,51–59] and Morocco [60–67]. The majority of these investigations involve inventories or inventory-based evaluations, which correspond to the initial stages of geoheritage conservation proposed by Brilha [9]. The evaluation methods used in these studies primarily adopt approaches developed by European researchers, including Brilha [9], Vujičić *et al.* [10] and Ruban *et al.* [7,24] directly, or use simple original methods with qualitative evaluations. Moreover, some studies have focused specifically on evaluating the endangerment status of geoheritage sites in arid environments [52,55]. In Oman and the UAE, Sayama *et al.* [68] found that 14% of Quaternary geoheritage sites were already destroyed, mainly due to quarrying and economic development.

Of studies from the MENA region, Siuki *et al.*'s study [69] on the evaluation of geosites in the Razavi Khorasan region in eastern Iran stands out as an exception. By using input from tourist and scientific specialists, this study developed an evaluation method focused on the touristic potential of geoheritage sites and used it to rank sites in Razavi Khorasan. Developed using input from multiple stakeholder groups, this method provides a high level of objectivity. On the other hand, the study offers limited instructions on how to apply the resulting method, most likely as it was designed for use at a local scale. Moreover, the criteria considered in this study differ significantly from any other methods. This may mean that the required criteria are different for different regions, but it makes comparison with other existing methods difficult. Given these considerations, further studies are required to examine the specificity of geoheritage evaluation in arid environments, in terms of weighting and criteria required.

### (d) Temporal/typological subdivision of geoheritage evaluation

Geoheritage includes many types of sites with a wide range of sizes, ages and processes. Habibi *et al.* [50] provide a list of 21 geoheritage types, such as stratigraphical, geomorphological, paleontological, igneous and palaeogeographical sites. Most methods for geoheritage evaluation target all types of geoheritage sites [6–10]. Comparatively, not many specialized methods have been developed for certain types of geoheritage sites, other than a few methods, such as Alcalá and Lockley's method [70] for dinosaur track sites.

An exception to this trend is geomorphological heritage sites, or geomorphosites. Geomorphosites are defined as ‘qualifying geomorphological sites that are part of the geomorphological heritage’ ([1], p. 14). Reynard [1] makes three main distinctions between general geoheritage sites and geomorphosites, which are as follows:

1. Aesthetic character: they are often defined by their beauty more so than other types of geoheritage sites, with many of them being considered natural monuments.
2. Dynamic nature: they are experiencing ongoing earth surface processes and current earth dynamics can be observed with these sites. The current state of these sites may be prone to change through natural processes such as weathering. Although geomorphosites may be damaged by these natural processes, they should not be interrupted to protect the current state of the sites.
3. Scale: they can be very large scale which can encompass entire landscapes, such as alluvial plains, or they can be smaller individual features.

In addition, as can be seen from the proposed use of the cultural geomorphology concept by Panizza & Piacente [71], geomorphological sites demonstrate a strong connection with human culture, whether it be archaeological, historical, architectural or beyond. Geomorphosites were formed, are forming, or will be formed, contemporaneously with the history of humans. This synchronicity provides geomorphosites the uniqueness as sites that provide records of the interactions of humans and the abiotic environment through time and document the impact of humans in this relationship. In arid environments, the use of this connection has helped many archaeological studies, to better understand topics such as human migration out of Africa [72], ancient-human responses to arid events in the Cusco region, Peru [73], and human settlement patterns in Australia [74]. Furthermore, some of these sites and/or records have evoked human creativity, in the sense of art, technology or cultural traditions, as exemplified by the religious and artistic significance of Mount Fuji in Japan [75,76].

In different parts of the world such as arid environments, however, the defining features of geomorphosites do not always hold true for all Quaternary geological or geomorphological sites, especially as these sites are not made distinct by their aesthetic character. As can be seen from figure 2, characteristic geomorphological sites in arid environments are not always majestic sites that could be considered natural monuments. This distinction is important, as many geoheritage sites are protected due to their aesthetic values, especially for the purpose of tourism [77], but that is not the primary criterion for the conservation of many others [2]. To make this distinction, this study will use the term Quaternary geoheritage, to refer to all geological/geomorphological sites from the Quaternary period, including geomorphosites.

Various evaluation methods have been developed targeting geomorphosites [3,19], including two of the four aforementioned popular methods [5,23] of geoheritage evaluation. Some distinguishing features of these methods are the inclusion of the aesthetic value and the cultural value as separate dimensions in the evaluation, which reflects the distinctive features of geomorphosites. In fact, Santos *et al.* [78], with a case study that compares a coastal temperate region of Brazil and the alpine region of the Alps, found significant differences in the evaluation outcomes between methods developed for general geoheritage sites and methods developed for geomorphosites. In the case of scientific values, the study pointed out the differences in the criteria considered, as the main reason for the differences in the evaluation outcomes. On the other hand, in a similar study in a temperate region of Brazil, Mucivuna *et al.* [18] found no significant differences in the outcomes between the general and specialized evaluation methods. This study suggested the use of existing evaluation methods in future research and advised to limit the use of specialized methods for only ‘specific features’. These studies do not provide a conclusive guideline, but it is noteworthy that arid environments have not been considered in such a context. Given their distinctive characteristics, Quaternary geoheritage sites from arid environments may need to be considered as ‘specific features’.

Overall, given the (a) lack of evaluation methods using input from a group of experts, (b) lack of evaluation methods targeting arid environments and (c) reported endangerment of Quaternary geoheritage sites identified in previous literature, this study aims to develop a new evaluation method for the scientific values of Quaternary geoheritage sites in arid environments by consulting a group of experts on the weighting strategy. In so doing, it also seeks to compare existing methods with this new method developed by the opinions of experts on arid environments.

## 2. Methods

To gather input required from experts on Quaternary geoscience of arid environments, an online questionnaire was created and distributed towards postgraduate students and academics in geosciences (i.e. geologists, geomorphologists, palaeontologists, physical geographers and hydrologists) or related disciplines that use geoscientific data (i.e. archaeologists, evolutionary anthropologists), with research interest in arid environments. The recruitment was conducted through geosciences and geoheritage conferences, social media adverts (see appendix i) and direct emails.

The survey asked researchers about:

1. Their profession
2. Their disciplinary expertise
3. Which criteria (out of the 10 as shown in table 1) should be included to evaluate the scientific value
4. The relative importance of criteria that should be included to evaluate the scientific value
5. The appropriateness of various proxies to evaluate criteria that should be included in the evaluation
6. Comments and suggestions

A 5-option scale, ranging from 1 to 5, was used for most of the questions (for the full questionnaire, see appendix ii) with the following description for each value:

1. Should not be ignored, but comparatively it is not very important
2. Should be considered to some degree
3. Should be considered
4. Very important to determine the scientific value
5. Essential, most important to determine the scientific value

The purpose of the study was to ask the participants to decide the relative importance of criteria used evaluate the scientific value of Quaternary geoheritage sites in arid environments. The criteria considered in the questionnaire were selected based on those used in past literature, with a couple of additions to reflect the characteristics of Quaternary geoheritage sites in arid environments. Most criteria used in past literature were selected, except for ones that overlap in its scope with other criteria. A criterion on the connection with the regional archaeology/evolutionary anthropology and another on the chronological resolution were added, as Quaternary geoscience research has made large contributions to these disciplines in arid regions around the world [73,74,79–84]. Table 1 describes the 10 criteria selected and outlines the criteria included in popular methods for geoheritage evaluation.

For the proxies, as many past evaluation systems rely on ‘expert opinions’, they do not provide clear proxies to quantify each factor. In order to understand which proxies are most suitable, the questionnaire asked participants to rate the most appropriate proxy from those used in the previous literature, such as the number of papers published on the site for scientific knowledge and the analysis of protection status for integrity, and potential proxies proposed by the author, such as the analysis of satellite imagery for integrity.

The weight for each criterion used to calculate the overall scientific value was determined using the mean of all responses. Numbers from the 5-point scale were used directly, but to reflect the opinions of those who answered against including a criterion in the assessment, a numerical value of −4 was given for the answer: ‘the criterion should not be included in the evaluation’. The value −4 was selected as it allows the mean of two responses, when one respondent considers a criterion as the most important (indicated by a 5), and another considers it as it ‘should not be included’, to equal the lowest value possible, 1, for the relative importance of a criterion.

## 3. Results

**Figure 3.**
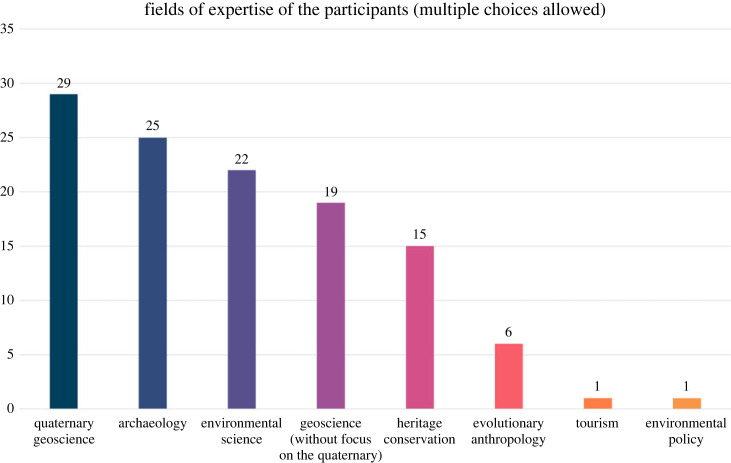
Fields of expertise of the participants of the questionnaire. (Online version in colour.)

The questionnaire was conducted between November 2021 and July 2022, and was answered by 49 researchers. The professions of participants include 6 PhD students, 42 academic researchers and 1 professional geoscientist. Of the academic researchers, 2 are also professional geoscientists, and 1 is a heritage manager. Their fields of expertise are outlined in figure 3. Of the 25 participants with archaeological expertise, 15 are also experts in Quaternary geoscience and 3 are either experts in wider geoscience or environmental science. Of the 20 participants without expertise in Quaternary geoscience, 10 are experts in archaeology, 7 in geosciences and 5 in heritage conservation.

**Figure 4.**
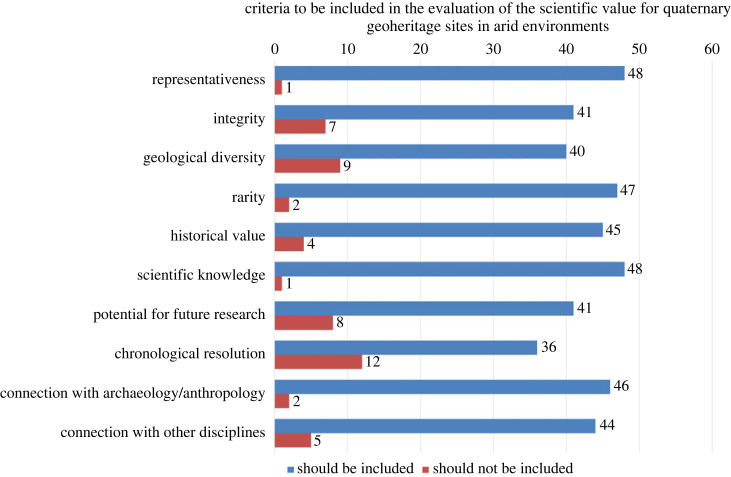
Number of responses to the questionnaire indicating the necessity of including each criterion in assessing the scientific value of Quaternary geoheritage sites in arid environments (*n* = 49, with respondents given the option to opt out of answering). (Online version in colour.)

The outcomes of the survey of the necessity and relative importance of each criterion are described in figures 3 and 4. For the necessity, none of the criteria were unanimously considered necessary, but representativeness and scientific knowledge were considered necessary by most participants with only one response against the inclusion of the criterion. This was followed by rarity and connection with archaeology/anthropology with two responses against their inclusion. Contrarily, chronological resolution, geological diversity and potential for future research received the most responses against inclusion, with 12, 9 and 8 participants against their inclusion, respectively.

As shown in table 2, for the relative importance of criteria, rarity had both the highest mean and median, followed by representativeness and scientific knowledge. These three criteria stood out as the most important, with more than a 0.5 difference in the mean relative importance to the next highest criterion, archaeological/anthropological connections. On the other end, chronological resolution had the lowest relative importance with a mean value of 1.65, about 0.47 lower than the second lowest criterion, geological diversity. In order to calculate the scientific value reflecting the relative importance of each criterion, a weighting system out of 100 was developed, as shown in table 2.

**Table 2.** Summary of the responses to the questionnaire on the relative importance of each criterion for the scientific evaluation of Quaternary geoheritage sites in arid environments (number in parentheses represent the number of answers).

							potential			connection
	represent-		geological		historical	scientific	for future	chronological	archaeo/anthro	with other
	ativeness	integrity	diversity	rarity	importance	knowledge	research	resolution	connection	disciplines
	(49)	(48)	(49)	(49)	(49)	(49)	(49)	(48)	(48)	(49)
mean	3.96	2.57	2.12	3.98	2.98	3.92	2.53	1.65	3.45	2.80
median	4	4	3	5	4	4	4	3	4	4
weight out of 100	13.22	8.60	7.09	13.29	9.95	13.08	8.45	5.50	11.51	9.33

Finally, the suitability of proxies for use as parameters for each criterion, as listed in table 3. Overall, site visits and expert judgement are consistently considered as one of the most appropriate proxies for the evaluation of the scientific value. Literature review and analysis scored relatively highly, especially for representativeness, rarity and historical significance. On the other hand, proxies related to publication (i.e. number of citations and number of publications) are considered as poor metrics, with a low relative importance value, especially those related to the number of citations. Beyond ‘discussions with experts', the highest rated proxy is generally those that have been used in previous methods. Proxies proposed by the author such as ‘comparative analysis of images of the site’ for representativeness and the ‘analysis of satellite imagery’ for integrity were not considered very appropriate.

**Table 3.** Summary of the responses to the questionnaire towards the suitability of proxies for the scientific evaluation of Quaternary geoheritage sites in arid environments (number in parentheses represent the number of answers).

	representativeness			integrity			geological diversity			rarity		historical significance			
	comparative analysis of literature (47)	comparative analysis of images of the site (44)	number of articles published on the site (45)	analysis of satellite imagery (41)	analysis of the protection status (40)	site visits (37)	literature review (38)	discussions with experts (32)	site visits (34)	literature review (46)	discussions with experts (41)	literature analysis (44)	discussions with experts (39)		
**mean**	3.80	3.56	2.32	3.41	3.53	4.70	3.61	3.88	4.53	4.11	4.22	4.09	4.15		
	scientific knowledge			future research			chronological resolution		connections with archaeology/anthropology				connections with other disciplines		
	number of citations (47)	publication in high-impact journals (48)	number of articles published on the site (48)	literature analysis and search for ongoing projects (41)	mentions of need for future research in academic literature (41)	discussions with experts (37)	depth of the sequence (34)	number of chronological records collected from the site (36)	chronological proximity or relationship with regional archaeological sites (46)	discussions with experts (44)	citation by archaeology or evolutionary anthropology articles (41)	citation in high-impact journal articles on archaeology or evolutionary anthropology (39)	citation by non-geoscience, archaeology, or anthropology, articles (43)	citation in high-impact journals (42)	discussions with experts (39)
**mean**	2.94	2.73	3.44	3.66	3.63	4.24	3.62	3.92	4.20	4.23	3.43	2.86	3.42	2.52	4.23

## 4. Discussion

The number of participants in this study, 49, is one of the highest for a study focused on developing an evaluation method for geoheritage sites. The number of participants, as well as their academic background focused on disciplines related to Quaternary geosciences, provides appropriate representation for the purpose of this study. Overall, the study has been able to reflect the opinion of a breadth of regional experts, but a limitation of approaching researchers as participants is the lack of representation of indigenous knowledge, which should be addressed in future studies.

To finalize the criteria and weighting system used to evaluate Quaternary geoheritage sites in arid environments, the necessity of each criterion must be considered. The criterion that stands out as unimportant in the result of this study is chronological resolution, the only one with a mean value below 2. This result is mostly because this criterion has been considered unnecessary by 25% of all participants. The relatively low importance of this criterion is apparent in the categorized data, as shown in table 2. Interestingly, even when excluding the ‘should not include’ answers, chronological resolution is the criterion with the second lowest relative importance. The chronological resolution criterion gained relatively high support from archaeologists and anthropologists both with and without expertise in Quaternary geoscience, most likely as they use the chronological data for their research or due to differences in semantic views between disciplines. Nevertheless, even in this case, chronological resolution was within the three lowest ranked criteria. Additionally, as noted by one of the participants, the chronological resolution criterion could possibly be considered within the scientific knowledge criterion. As mentioned in previous studies [15,85], overlapping criteria should be combined, when possible, to simplify the evaluation. Therefore, the chronological resolution criterion was removed entirely from the final evaluation method.

The final weighting system developed by this study is compared to other methods in table 5. The main characteristics of the method developed in this study include the relatively high number of criteria, lower importance of representativeness and historical value, higher importance of scientific knowledge, and the inclusion of criteria on connections with archaeology/anthropology and with other elements. The higher number of criteria can be explained by the study's approach to include most criteria used in previously developed methods, and the outcome of the questionnaire that indicated most criteria as necessary for the evaluation. As discussed previously, having many criteria can lead to complexity in analysis and overlap in assessment. Potential overlaps for this method include scientific knowledge and historical value, as seen by the fact that only Brilha [9] uses these two criteria together in the assessment. When implementing this method, statistical analyses, like those conducted by Bruschi *et al.* [15] may be necessary to reduce the number of criteria used to ensure that there is no redundancy in the assessment.

**Table 4.** Differences in the weighting for the scientific value assessment in different scenarios.

								potential			
								for			connection
				geological		historical	scientific	future	chronological	archaeo/anthro	with other
		representativeness	integrity	diversity	rarity	importance	knowledge	research	resolution	connection	disciplines
all responses	mean (all criteria)	3.96	2.57	2.12	3.98	2.98	3.92	2.53	1.65	3.45	2.80
	weight (all criteria)	13.2	8.6	7.1	13.3	9.9	13.1	8.4	5.5	11.5	9.3
	weight (without chronological resolution)	*14*.*0*	*9*.*1*	*7*.*5*	*14*.*1*	*10*.*5*	*13*.*8*	*8*.*9*	*—*	*12*.*2*	*9*.*9*
without ‘should not include’	mean (all criteria)	4.13	3.73	3.50	4.32	3.60	4.09	3.80	3.53	3.78	3.57
	weight (all criteria)	10.8	9.8	9.2	11.4	9.5	10.7	10.0	9.3	9.9	9.4
	weight (without chronological resolution)	12.0	10.8	10.1	12.5	10.4	11.8	11.0	—	10.9	10.3
without archaeologists and anthropologists with no expertise in quaternary geosciences	mean (all criteria)	4.08	2.15	2.08	3.85	2.85	3.62	2.38	1.44	2.95	2.67
	weight (all criteria)	14.5	7.7	7.4	13.7	10.1	12.9	8.5	5.1	10.5	9.5
	weight (without chronological resolution)	15.3	8.1	7.8	14.5	10.7	13.6	9.0	—	11.1	10.0
only quaternary geoscientists	mean (all criteria)	4.03	1.59	1.45	3.72	2.66	3.83	2.28	2.24	2.90	2.59
	weight (all criteria)	14.8	5.8	5.3	13.7	9.7	14.0	8.3	8.2	10.6	9.5
	weight (without chronological resolution)	16.1	6.3	5.8	14.9	10.6	15.3	9.1	—	11.6	10.3

**Table 5.** Comparison of percentage for each criterion used in weighting methods for the assessment of scientific values for geoheritage sites.

		Bruschi *et al.* [15]				current study
		(weight for criteria			current	adapted to
	Brilha [9]	related to scientific value)	Pereira & Pereira [23]	Reynard *et al.* [5]	study	Reynard *et al.* [5]
representativeness (%)	30	21.6 (good example of process)	18.2	25	14	27.8
integrity (%)	15	17.0 (fragility)	18.2	25	9.1	20.9
geological diversity (%)	5	n.a.	18.2	n.a.	7.5	n.a.
rarity (%)	15	21.2	27.3 (rarity at national level + rarity at regional level)	25	14.1	28.5
historical value (%)	20 (key locality)	n.a.	n.a.	25 (palaeogeographical value)	10.5	22.8
scientific knowledge (%)	5	12.6 (degree of knowledge)	9.1	n.a.	13.8	n.a.
potential for future research (%)	n.a.	n.a.	n.a.	n.a.	8.9	n.a.
archaeo/anthro connection (%)	n.a.	16.4 (cultural interest)	n.a.	n.a.	12.2	n.a.
connection with other disciplines (%)	n.a.	12.2 (related to human issues)	n.a.	n.a.	9.9	n.a.
others (%)	10 (use limitation)	n.a.	9.1 (non-geomorphological geodiversity)	n.a.	n.a.	n.a.

Among the weight of the criteria, the high relative importance of archaeological/anthropological connections is noteworthy, as it is a criterion that has not been discussed in previous evaluation methods. As can be seen from comparing the results from all responses to the results from only Quaternary geoscientists in table 4, this criterion was (rather predictably) considered more important by archaeologists and anthropologists. Nevertheless, in all categories of participants considered in table 4, its relative importance is ranked either fourth or fifth and only two participants considered it unnecessary in assessing the overall scientific value. The importance of this connection is emphasized by the relatively lower evaluation of the importance of the connections with other disciplines as well. These outcomes help justify the use of a specialized evaluation method for the scientific values of Quaternary geoheritage sites in arid environments.

Another strength of the outcome of this study is its compatibility with other methods. As the criteria considered in this study encompass most criteria from previous studies, it can easily be adapted to create a derivative method that reflects the criteria used in a certain study, but with a weight system that reflects regional experts. For example, if a researcher wishes to use only the four criteria used by Reynard *et al.* [5] in the assessment, the relative importance of those criteria from this study could be selected to create a new weighting system, as presented in table 5. The resulting weighting system includes a 7.6% difference between the most important and the least important criterion, reflecting the regional experts' preferences towards a method that originally uses equal weight for all criteria. As this study reflects the preferences of many regional experts, such a partial application may be useful to reduce subjectivity in existing methods.

Using the responses to the questionnaire, appropriate proxies used to assess each criterion were determined, as described in table 6. The responses to the questionnaire highlighted the difficulty of assessing every criterion using purely quantitative parameters, with the ‘discussions with experts’ proxy rated the highest for five out of six criteria for which it was an option. This proxy was considered more appropriate than proxies that can be assessed quantitatively, such as ‘number of publications', ‘number of citations’ or ‘literature analysis’. For the use of proxies related to the academic literature, three participants expressed their concerns, pointing out shortfalls such as the prioritization of older sites, the exclusion of information gained from grey literature, and the exclusion of sites that have not yet been studied. An expert judgement may be necessary to consider newer sites or sites that have been understudied or have never been studied. Criteria that may require such an input from experts are highlighted with an asterisk in table 6. In developing the list of appropriate proxies, as no new quantitative proxy gained strong support, for criteria that have been assessed previously using a parametric system, proxies suggested in previous literature [9,26,28] were used. For criteria that have not been assessed with proxies in previous studies (i.e. future research, connections with archaeology/anthropology, connections with other disciplines), the highest ranked quantitative proxy was selected. For the evaluation of all criteria, the 4-scale point system (0, 1, 2, 4 points) developed by Brilha [9] was adopted.

**Table 6.** List of proxies and points system to be used in evaluating each criterion.

	0 point	1 point	2 points	4 points
representativeness	little use as a model to represent, even partially, a feature or process	the site reasonably illustrates elements or processes in the study area,	the site is a good example in the study area to illustrate elements or processes	the site is the best example in the study area to illustrate elements or processes
integrity	strongly degraded: the site is practically destroyed	site with preservation problems and with the main geological elements quite altered or modified	site not so well preserved, but the main geological elements are still preserved	the main geological/geomorphological elements are very well preserved
geological diversity	sites with only one type of distinct geological/geomorphological features with scientific relevance	site with two types of distinct geological/geomorphological features with scientific relevance	site with three types of distinct geological/geomorphological features with scientific relevance	site with more than three types of distinct geological/geomorphological features with scientific relevance
rarity	there are more than five examples of similar sites in the study area	in the study area, there are four to five examples of similar sites	in the study area, there are two to three examples of similar sites	the site is the only occurrence of this type in the study area
historical significance	it does not meet the following three criteria	the site is used by national science, directly related with the geological framework under consideration	the site is used by international science, directly related with the geological framework under consideration	the site is recognized as a GSSP or ASSP by the IUGS or is an IMA reference site
scientific knowledge^a^	there are no published studies, conference abstracts or doctoral theses on the site	there are abstracts presented in international scientific events about this site, directly related with the geological framework under consideration	there are papers in national scientific publications about this site, directly related with the geological framework under consideration	there are papers in international scientific journals about this site, directly related with the geological framework under consideration
future research^a^	no ongoing or planned projects on the site, or mention of potential for future research in academic and/or grey literature	one mention of potential for future research at the site, but no ongoing project	multiple mentions of potential for future research at the site and/or a plan or proposals to conduct research at the site	project(s) ongoing currently at the site
connections with archaeo/anthro^a^	no data from the site, or no data that correspond with archaeological or anthropological records	data from site have been used in a single archaeological or anthropological study at the local level	data from site have been used in multiple archaeological or anthropological studies at local level	data from site have been used in archaeological or anthropological studies at the regional/global level
connections with other disciplines^a^	no citation towards studies on the site by disciplines other than geosciences, archaeology or anthropology	single citation towards studies on the site by disciplines other than geosciences, archaeology or anthropology	two or three citations toward studies on the site by disciplines other than geosciences, archaeology or anthropology	more than three citations toward studies on the site by disciplines other than geosciences, archaeology or anthropology

^a^Criteria that may need expert input if the site is new, understudied or have never been studied (adapted from Brilha [9] and García-Cortéz *et al.* [28]).

Finally, this study highlights the necessity for future studies to assess the need for specialized methods in geoheritage values beyond the scientific value. Regional experts in different additional values of geoheritage (touristic, educational, etc.) must be consulted to understand whether the previously developed methods require adjustments in different regions. A need for a modified method is likely, especially as this study identified connection with archaeology/anthropology, a criterion with strong cultural and human elements, as a highly necessary criterion in evaluating the scientific value. Similar cultural dimensions of geoheritage would likely influence the way that more human-centric values are assessed. In conducting future studies to develop new methods for these additional values, a more localized geographical focus may be necessary, as socioeconomic and cultural heterogeneity would likely lead to differences in preferences.

## 5. Conclusion

This study assessed the necessity of a specialized evaluation method for the scientific values of Quaternary geoheritage sites in arid environments by using input from scientific experts from Quaternary geosciences and related fields. The analysis of the responses from 49 researchers demonstrated differences in the preferences towards the relative importance of criteria used in the evaluation compared to those indicated in previously developed methods designed for application around the world.

The specialized method developed in this study addressed this issue by selecting the criteria and their respective weights based on the responses by regional experts. The resulting method includes key differences such as lower weight for representativeness, integrity and historical value, higher weight for scientific knowledge, and the inclusion of two specialized criteria, connections with archaeology/anthropology and connections with other disciplines. This study also explored the appropriateness of proxies for evaluating each criterion. The participants' responses highlighted the challenges of relying solely on quantitative parameters for assessment, with expert judgement consistently considered as highly appropriate.

In practical application, adjustments to this method may be necessary to reduce the relatively high number of criteria considered to reduce complexity and overlap in assessment. Nevertheless, the results of this study can be used not only as a fully new method, but flexibly as a tool to create versions of existing methods with a region-specific weighting system. By selecting the preferences of regional experts for the criteria required for the method of choice, a revised method with considerations for the arid environment can easily be created to help reduce subjectivity and improve applicability.

Overall, the result of this study suggests the need to consider the necessity of diversity in methods for geoheritage evaluation not only for Quaternary geoheritage sites in arid environments, but for other environments and regions as well. Consistency in evaluation methods is desirable, but if consistency hinges on the quality of the evaluation, it should not be the primary concern. Even for Quaternary geoheritage sites in arid environments, evaluation methods with a narrower focus may be necessary to capture subtle differences in the evaluation. The method presented in this study is the outcome of an effort to achieve the right balance between the regional and environmental differences and the need for comparability and consistency in evaluation methods. Such considerations are required for regional and environmental differences in geoheritage values beyond the scientific domain as well. Future research is encouraged to assess the need for specialized evaluation methods in different environments at different scales, and for values not limited to the scientific value, such as educational value and touristic value, involving input from regional experts in respective fields.

## Data Availability

The raw data used of the questionnaire answers (except for personal data), a copy of the questionnaire and the advertisement poster are available as electronic supplementary materials [86].

## References

[1] Reynard E. 2009 The assessment of geomorphosites. In Geomorphosites (eds E Reynard, P Coratza, G Regolini-Bissig), pp. 63-71. Munich, Germany: Verlag Dr. Friedrich Pfeil.

[2] Brilha J. 2018 Chapter 4 - geoheritage: inventories and evaluation. In Geoheritage (eds E Reynard, J Brilha), pp. 69-85. Amsterdam, The Netherlands: Elsevier.

[3] Coratza P, Giusti C. 2005 Methodological proposal for the assessment of the scientific quality of geomorphosites. Ital. J. Quat. Sci. **18**, 307-313.

[4] Panizza M. 2001 Geomorphosites: concepts, methods and examples of geomorphological survey. Chin. Sci. Bull. **46**(S1), 4-5. (10.1007/BF03187227)

[5] Reynard E, Fontana G, Kozlik L, Scapozza C. 2007 A method for assessing the scientific and additional values of geomorphosites. Geographica Helvetica **62**, 148-158. (10.5194/gh-62-148-2007)

[6] Knapik R, Jała Z, Sobczyk A, Migoń P, Aleksandrowski P, Szuszkiewicz A, Krąpiec M, Madej S, Krakowski K. 2009 Inwentaryzacja i waloryzacja geostanowisk Karkonoskiego Parku Narodowego i jego otuliny oraz wykonanie mapy geologicznej tego obszaru. Jelenia Góra. 5-8.

[7] Ruban DA. 2010 Quantification of geodiversity and its loss. Proc. Geol. Assoc. **121**, 326-333. (10.1016/j.pgeola.2010.07.002)

[8] De Lima FF, Brilha JB, Salamuni E. 2010 Inventorying geological heritage in large territories: a methodological proposal applied to Brazil. Geoheritage **2**, 91��99. (10.1007/s12371-010-0014-9)

[9] Brilha J. 2016 Inventory and quantitative assessment of geosites and geodiversity sites: a review. Geoheritage **8**, 119-134. (10.1007/s12371-014-0139-3)

[10] Vujičić MD, Vasiljević DA, Marković SB, Hose TA, Lukić T, Hadžić O, Janićević S. 2011 Preliminary geosite assessment model (gam) and its application on Fruška gora mountain, potential geotourism destination of Serbia. Acta Geogr. Sloven. **51**, 361-376. (10.3986/AGS51303)

[11] Siuki HS, Kowalczyk A. 2012 Criteria of selection by potential tourists of destinations of geotourism value (based on the example of the region of Khorasan, Iran). Problems of Tourism and Recreation **3**, 41-57.

[12] Brocx M, Semeniuk V. 2007 Geoheritage and geoconservation-history, definition, scope and scale. J. R. Soc. Western Austr. **90**, 53-87.

[13] UNESCO 2021 Operational guidelines for the implementation of the world heritage convention. Paris, France: UNESCO.

[14] Council NC. 1990 Earth science conservation in Great Britain: A strategy. Peterborough, UK: Nature Conservancy Council.

[15] Bruschi VM, Cendrero A, Albertos JAC. 2011 A statistical approach to the validation and optimisation of geoheritage assessment procedures. Geoheritage **3**, 131-149. (10.1007/s12371-011-0038-9)

[16] Brocx M, Semeniuk V. 2015 Using the Geoheritage Tool-Kit to identify inter-related geological features at various scales for designating geoparks: case studies from Western Australia. In From geoheritage to geoparks: case studies from Africa and beyond (eds E Errami, M Brocx, M Semeniuk), pp. 245-259. Cham, Switzerland: Springer.

[17] Bruschi V, Cendrero A. 2009 Direct and parametric methods for the assessment of geosites and geomorphosites. Geomorphosites **9**, 73-88.

[18] Mucivuna VC, Motta Garcia MDG, Reynard E. 2022 Comparing quantitative methods on the evaluation of scientific value in geosites: analysis from the Itatiaia National Park, Brazil. Geomorphology **396**, 107988. (10.1016/j.geomorph.2021.107988)

[19] Kubalìková L. 2013 Geomorphosite assessment for geotourism purposes. Czech J. Tourism **2**, 80-104. (10.2478/cjot-2013-0005)

[20] Pralong J-P. 2005 A method for assessing tourist potential and use of geomorphological sites. Géomorphologie: relief, processus, environnement. **11**, 189-196. (10.4000/geomorphologie.350)

[21] Zwoliński Z, Najwer A, Giardino M. 2018 Methods for assessing geodiversity. In Geoheritage: Assessment, Protection, and Management (eds E Reynard, J Brilha), pp. 27-52. Amsterdam, The Netherlands: Elsevier.

[22] Erhartič B. 2010 Geomorphosite assessment. Acta Geogr. Sloven. **50**, 295-319. (10.3986/AGS50206)

[23] Pereira P, Pereira D. 2010 Methodological guidelines for geomorphosite assessment. Géomorphologie: relief, processus, environnement **16**, 215-222. (10.4000/geomorphologie.7942)

[24] Ruban DA, Sallam ES, Khater TM, Ermolaev VA. 2021 Golden triangle geosites: preliminary geoheritage assessment in a geologically rich area of Eastern Egypt. Geoheritage. **13**, 54. (10.1007/s12371-021-00582-8)

[25] Zorlu K, Dede V. 2023 Assessment of glacial geoheritage by multi-criteria decision making (MCDM) methods in the Yalnızçam Mountains, Northeastern Türkiye. Int. J. Geoherit. Parks **11**, 100-117. (10.1016/j.ijgeop.2023.01.001)

[26] Pereira P, Pereira D, Caetano Alves MI. 2007 Geomorphosite assessment in Montesinho Natural Park (Portugal). Swiss J. Geogr. **62**, 159. (10.5194/gh-62-159-2007)

[27] Solarska A, Jary Z. 2010 Geoheritage and geotourism potential of the Strzelin Hills (Sudetic Foreland, SW Poland). Geogr. Pannonica. **14**, 118-125. (10.5937/GeoPan1004118S)

[28] García-Cortés A, Vegas J, Carcavilla L, Díaz-Martínez E. 2019 Conceptual base and methodology of the Spanish Inventory of Sites of Geological Interest (IELIG). Madrid, Spain: Instituto Geológico y Minero de España, p. 102.

[29] Brocx M, Semeniuk V. 2009 Developing a tool-kit for geoheritage and geoconservation in Western Australia. ProGeo Newsletter **1**, 5-9.

[30] Suzuki DA, Takagi H. 2018 Evaluation of geosite for sustainable planning and management in geotourism. Geoheritage **10**, 123-135. (10.1007/s12371-017-0225-4)

[31] Woo KS, Kim L. 2018 Geoheritage evaluation of caves in Korea: a case study of limestone caves. In Geoheritage (eds E Reynard, J Brilha), pp. 373-386. Amsterdam, The Netherlands: Elsevier.

[32] Santos DS, Mansur KL, Seoane JCS, Mucivuna VC, Reynard E. 2020 Methodological proposal for the inventory and assessment of geomorphosites: an integrated approach focused on territorial management and geoconservation. Environ. Manage. **66**, 476-497. (10.1007/s00267-020-01324-2) 32632499

[33] Bruschi VM, Cendrero A. 2005 Geosite evaluation; can we measure intangible values? Alp. Medit. Quat. **18**, 293-306.

[34] Sillitoe P. 2009. In Local science Vs global science approaches to indigenous knowledge in international development (ed. P Sillitoe), 1st edn. Brooklyn, NY: Berghahn Books.

[35] Whyte KP, Brewer JP, Johnson JT. 2016 Weaving Indigenous science, protocols and sustainability science. Sustainability Sci. **11**, 25-32. (10.1007/s11625-015-0296-6)

[36] Mortimore M *et al.* 2009 Dryland opportunies: a new paradigm for people, ecosystems and development. London, UK: International Union for Conservation of Nature (IUCN).

[37] Thomas DSG. 2011 Arid zone geomorphology, 3rd edn. New York, NY: Wiley.

[38] Salamzadeh A, Ebrahimi P, Soleimani M, Fekete-Farkas M. 2021 An AHP approach to identify the barriers of sustainable geotourism development in Iran: an economic view. Geoheritage. **13**, 65. (10.1007/s12371-021-00581-9)

[39] Moradipour F, Moghimi E, Beglou MJ, Yamani M. 2020 Assessment of urban geomorphological heritage for urban geotourism development in Khorramabad City, Iran. Geoheritage **12**, 40. (10.1007/s12371-020-00466-3)

[40] Soltan BH. 2021 Characteristic features of the proposed Jabal Sanam Geopark Southern Iraq. Geoheritage **13**, 77. (10.1007/s12371-021-00599-z)

[41] Moradi A, Maghsoudi M, Moghimi E, Yamani M, Rezaei N. 2021 A comprehensive assessment of geomorphodiversity and geomorphological heritage for Damavand Volcano Management, Iran. Geoheritage **13**, 39. (10.1007/s12371-021-00551-1)

[42] Tomić N, Sepehriannasab B, Marković SB, Hao Q, Lobo HAS. 2021 Exploring the preferences of Iranian Geotourists: case study of shadows canyon and Canyon of Jinns. Sustainability **13**, 798. (10.3390/su13020798)

[43] Mehdipour Ghazi J, Hamdollahi M, Moazzen M. 2021 Geotourism of mining sites in Iran: An opportunity for sustainable rural development. Int. J. Geoherit. Parks **9**, 129-142. (10.1016/j.ijgeop.2021.02.004)

[44] Sadry BN, Mohammadi-Aragh A, Fehrest F, Bayatani A, Haji-Moradi A. 2021 Identifying and assessing geodiversities around Takht-e Soleyman World Heritage site to propose the territory as the third Geopark in Iran. In Global geographical heritage, geoparks and geotourism: geoconservation and development (eds RB Singh, D Wei, S Anand), pp. 15-42. Singapore: Springer Singapore.

[45] Khoshraftar R, Farsani NT. 2022 An introduction to geotourism of the Mahneshan county. Geoheritage. **14**, 28. (10.1007/s12371-022-00671-2)

[46] Molchanova TK, Ruban DA. 2019 New evidence of the Bangestan Geoheritage resource in Iran: beyond hydrocarbon reserves. Resources **8**, 35. (10.3390/resources8010035)

[47] Pourahmad A, Hosseini A, Pourahmad A, Zoghi M, Sadat M. 2018 Tourist value assessment of geotourism and environmental capabilities in Qeshm Island, Iran. Geoheritage. **10**, 687-706. (10.1007/s12371-017-0273-9)

[48] Mehdipour Ghazi J, Audra P. 2022 Travertine Park in Azarshahr (NW Iran): an opportunity for geoheritage conservation and diminishing geohazards risk. Geoheritage. **14**, 99. (10.1007/s12371-022-00734-4)

[49] Farsani NT, Esfahani MAG, Shokrizadeh M. 2019 Understanding tourists’ satisfaction and motivation regarding mining geotours (Case Study: Isfahan, Iran). Geoheritage **11**, 681-688. (10.1007/s12371-018-0318-8)

[50] Habibi T, Ponedelnik AA, Yashalova NN, Ruban DA. 2018 Urban geoheritage complexity: evidence of a unique natural resource from Shiraz city in Iran. Resour. Policy **59**, 85-94. (10.1016/j.resourpol.2018.06.002)

[51] Plyusnina EE, Sallam ES, Ruban DA. 2016 Geological heritage of the Bahariya and Farafra oases, the central Western Desert, Egypt. J. Afr. Earth. Sci. **116**, 151-159. (10.1016/j.jafrearsci.2016.01.002)

[52] Abdelmaksoud KM, Al-Metwaly WM, Ruban DA, Yashalova NN. 2018 Geological heritage under strong urbanization pressure: El-Mokattam and Abu Roash as examples from Cairo, Egypt. J. Afr. Earth. Sci. **141**, 86-93. (10.1016/j.jafrearsci.2018.02.008)

[53] Sallam ES, Abd El-Aal AK, Fedorov YA, Bobrysheva OR, Ruban DA. 2018 Geological heritage as a new kind of natural resource in the Siwa Oasis, Egypt: the first assessment, comparison to the Russian South, and sustainable development issues. J. Afr. Earth. Sci. **144**, 151-160. (10.1016/j.jafrearsci.2018.04.008)

[54] Sallam ES, Fathy EE, Ruban DA, Ponedelnik AA, Yashalova NN. 2018 Geological heritage diversity in the Faiyum Oasis (Egypt): a comprehensive assessment. J. Afr. Earth. Sci. **140**, 212-224. (10.1016/j.jafrearsci.2018.01.010)

[55] Abdelmaksoud KM, Al-Metwaly WM, Ruban DA, Yashalova NN. 2019 Sand dune migration as a factor of geoheritage loss: evidence from the Siwa Oasis (Egypt) and implications for geoheritage management. Proc. Geol. Assoc. **130**, 599-608. (10.1016/j.pgeola.2019.07.001)

[56] Sallam ES, Ruban DA, Mostafa MT, Elkhodery MK, Alwilily RL, Molchanova TK, Zorina SO. 2020 Unique desert caves as a valuable geological resource: first detailed geological heritage assessment of the Sannur Cave, Egypt. Arab. J. Geosci. **13**, 141. (10.1007/s12517-020-5176-4)

[57] Sallam ES, Abd El-Samee MA, Bobrysheva OR, Yashalova NN, Ruban DA. 2020 Geological heritage of Luxor and its vicinities, Egypt: a new assessment and geotourism perspectives. Arab. J. Geosci. **13**, 76. (10.1007/s12517-019-5038-0)

[58] Kharbish S, Henaish A, Zamzam S. 2020 Geodiversity and geotourism in Greater Cairo area, Egypt: implications for geoheritage revival and sustainable development. Arab. J. Geosci. **13**, 451. (10.1007/s12517-020-05457-w)

[59] Khalaf EEDAH. 2022 Karst heritage as a tourist attraction: a case study in the White Desert National Park, Western Desert, Egypt. Geoheritage **14**, 94. (10.1007/s12371-022-00727-3)

[60] Mehdioui S, Hadi HE, Tahiri A, Haibi HE, Tahiri M, Zoraa N, Hamoud A. 2022 The Geoheritage of Northwestern Central Morocco Area: inventory and quantitative assessment of geosites for geoconservation, geotourism, geopark purpose and the support of sustainable development. Geoheritage **14**, 86. (10.1007/s12371-022-00712-w)

[61] Arrad TY, Errami E, Ennih N, Brahim O, El Mostafa E, Said B. 2020 From geoheritage inventory to geoeducation and geotourism implications: insight from Jbel Amsittene (Essaouira province, Morocco). J. Afr. Earth. Sci. **161**, 103656. (10.1016/j.jafrearsci.2019.103656)

[62] Mehdioui S, El Hadi H, Tahiri A, Brilha J, El Haibi H, Tahiri M. 2020 Inventory and Quantitative Assessment of Geosites in Rabat-Tiflet Region (North Western Morocco): preliminary study to evaluate the potential of the area to become a geopark. Geoheritage **12**, 35. (10.1007/s12371-020-00456-5)

[63] Berred S, Fadli D, El Wartiti M, Zahraoui M, Berred K, Sadki R. 2019 Geomorphosites of the semi-arid tata region: valorization of an unknown geoheritage for geotourism sustainable development (Anti-Atlas, South Morocco). Geoheritage **11**, 1989-2004. (10.1007/s12371-019-00414-w)

[64] Beraaouz M, Macadam J, Bouchaou L, Ikenne M, Ernst R, Tagma T, Masrour M. 2019 An inventory of geoheritage sites in the Draa Valley (Morocco): a contribution to promotion of geotourism and sustainable development. Geoheritage **11**, 241-255. (10.1007/s12371-017-0256-x)

[65] Bouzekraoui H, Barakat A, Mouaddine A, El Youssi M, Touhami F, Hafid A. 2018 Mapping geoheritage for geotourism management, a case study of Aït Bou Oulli Valley in Central High-Atlas (Morocco). Environ. Earth Sci. **77**, 1-15. (10.1007/s12665-018-7589-x)

[66] Arrad TY, Errami E, Ennih N. 2018 From scientific inventory to socio-economic sustainable development: Tidzi Diapir geosite (Essaouira basin, Morocco). J. Chem. Biol. Phys. Sci. **9**, 1-17. (10.24214/jcbps.D.9.1.00117)

[67] Bouzekraoui H, Barakat A, El Youssi M, Touhami F, Mouaddine A, Hafid A. 2018 Mapping geosites as gateways to the geotourism management in Central High-Atlas (Morocco). Quaestiones geographicae **37**, 87-102. (10.2478/quageo-2018-0007)

[68] Sayama K, Parker AG, Parton A, Viles H. 2022 Developing a geocultural database of quaternary palaeoenvironmental sites and archaeological sites in Southeast Arabia: inventory, endangerment assessment, and a roadmap for conservation. Sustainability **14**, 14096. (10.3390/su142114096)

[69] Siuki HS, Kowalczyk A, Atefeh E. 2012 A tourism demand based method of geosites assessment on geotourism prioritization modeling: the case of Razavi Khorasan Province. J. Hospital. Manag. Tourism **3**, 82-94. (10.5897/JHMT12.009)

[70] Alcalá L, Lockley M, Cobos A, Mampel L, Royo-Torres R. 2016 Evaluating the dinosaur track record: an integrative approach to understanding the regional and global distribution, scientific importance, preservation, and management of tracksites. In Dinosaur Tracks: The Next Steps (eds PL Falkingham, D Marty, A Richter), pp. 101-111. Bloomington, IN: Indiana University Press.

[71] Panizza M, Piacente S. 2003 Geomorfologia culturale. Bologna, Italy: Pitagora Editrorice.

[72] Nicholson SL, Hosfield R, Groucutt HS, Pike AWG, Fleitmann D. 2021 Beyond arrows on a map: the dynamics of Homo sapiens dispersal and occupation of Arabia during Marine Isotope Stage 5. J. Anthropol. Archaeol. **62**, 101269. (10.1016/j.jaa.2021.101269)

[73] Chepstow-Lusty A, Frogley MR, Bauer BS, Bush MB, Herrera AT. 2003 A late Holocene record of arid events from the Cuzco region, Peru. J. Quat. Sci. Publ. Quat. Res. Assoc. **18**, 491-502. (10.1002/jqs.770)

[74] Williams AN, Veth P, Steffen W, Ulm S, Turney CSM, Reeves JM, Phipps SJ, Smith M. 2015 A continental narrative: human settlement patterns and Australian climate change over the last 35,000 years. Quat. Sci. Rev. **123**, 91-112. (10.1016/j.quascirev.2015.06.018)

[75] Oguchi T, Oguchi CT. 2010 Mt. Fuji: the beauty of a symmetric stratovolcano. In Geomorphological landscapes of the world (eds P Migoń), pp. 303-309. The Netherlands: Springer.

[76] Chakraborty A, Jones TE. 2018 Mount Fuji: the volcano, the heritage, and the mountain. In Natural heritage of Japan: geological, geomorphological, and ecological aspects (eds A Chakraborty, K Mokudai, M Cooper, M Watanabe, S Chakraborty), pp. 167-175. Cham, Switzerland: Springer International Publishing.

[77] Chylińska D. 2019 The role of the picturesque in geotourism and iconic geotourist landscapes. Geoheritage **11**, 531-543. (10.1007/s12371-018-0308-x)

[78] Santos DS, Reynard E, Mansur KL, Seoane JCS. 2019 The specificities of geomorphosites and their influence on assessment procedures: a methodological comparison. Geoheritage **11**, 2045-2064. (10.1007/s12371-019-00411-z)

[79] Field JH, Dodson JR, Prosser IP. 2002 A Late Pleistocene vegetation history from the Australian semi-arid zone. Quat. Sci. Rev. **21**, 1023-1037. (10.1016/S0277-3791(01)00057-9)

[80] Madella M, Fuller DQ. 2006 Palaeoecology and the Harappan Civilisation of South Asia: a reconsideration. Quat. Sci. Rev. **25**, 1283-1301. (10.1016/j.quascirev.2005.10.012)

[81] Migowski C, Stein M, Prasad S, Negendank JF, Agnon A. 2006 Holocene climate variability and cultural evolution in the Near East from the Dead Sea sedimentary record. Quat. Res. **66**, 421-431. (10.1016/j.yqres.2006.06.010)

[82] Clarke J *et al.* 2016 Climatic changes and social transformations in the Near East and North Africa during the ‘long'4th millennium BC: a comparative study of environmental and archaeological evidence. Quat. Sci. Rev. **136**, 96-121. (10.1016/j.quascirev.2015.10.003)

[83] Goebel T, Hockett B, Rhode D, Graf K. 2021 Prehistoric human response to climate change in the Bonneville basin, western north America: The Bonneville Estates Rockshelter radiocarbon chronology. Quat. Sci. Rev. **260**, 106930. (10.1016/j.quascirev.2021.106930)

[84] Morgan C, Jamsranjav B, Tumurbaatar T, Barton L. 2022 Paleolakes, archaeology, and late Quaternary paleoenvironments in northwestern Mongolia. Quat. Res. **109**, 1-15. (10.1017/qua.2022.9)

[85] Mucivuna VC, Reynard E, Garcia M. 2019 Geomorphosites assessment methods: comparative analysis and typology. Geoheritage **11**, 1799-1815. (10.1007/s12371-019-00394-x)

[86] Sayama K. 2024 Promoting diversity in geoheritage evaluation: creating an evaluation method for the scientific value of Quaternary sites in arid environments. Figshare. (10.6084/m9.figshare.c.7031253)38342218

